# Clinical efficacy of highly agglutinative staphylococcin combined with neoadjuvant chemotherapy on patients with intermediate or advanced oral cancer

**DOI:** 10.12669/pjms.38.7.5323

**Published:** 2022

**Authors:** Nan Guo, Gengbing Lin, Xie Shi

**Affiliations:** 1 Nan Guo, Department of Stomatology, Fujian Provincial People’s Hospital, Affiliated People’s Hospital of Fujian University of Traditional Chinese Medicine, Fuzhou 350004, Fujian, China; 2 Gengbing Lin, Department of Stomatology, Fujian Provincial People’s Hospital, Affiliated People’s Hospital of Fujian University of Traditional Chinese Medicine, Fuzhou 350004, Fujian, China; 3 Xie Shi, Department of Orthodontics, Fujian Key Laboratory of Oral Diseases & Fujian Provincial Engineering Research Center of Oral Biomaterial & Stomatological Key lab of Fujian College and University, School and Hospital of Stomatology, Fujian Medical University & Institute of Stomatology, Fujian Medical University, Fuzhou 350004, Fujian, China

**Keywords:** Highly agglutinative staphylococcin, Chemotherapy, Intermediate or advanced oral cancer, Treatment

## Abstract

**Objectives:**

To evaluate the clinical efficacy of highly agglutinative staphylococcin combined with neoadjuvant chemotherapy on patients with intermediate or advanced oral cancer.

**Methods:**

A total of 80 patients with intermediate or advanced oral cancer treated in Affiliated People’s Hospital of Fujian University of Traditional Chinese Medicine from January 2020 to January 2022 were included. Patients were divided into two groups based on their treatment choice, with 40 cases in each group. Patients in the control group were given paclitaxel combined with cisplatin chemotherapy regimen: paclitaxel 150 mg/m2 and cisplatin 100 mg/m2 on Day-1, with 28 days as one cycle of chemotherapy for a total of three cycles. Patients in the experimental group received intramuscular injection of 500U highly agglutinative staphylococcin once a day for two weeks on the basis of chemotherapy, and continued the next course of treatment after 1 week of withdrawal, with a total of two courses. After treatment, the therapeutic effect, adverse drug reactions, changes in tumor markers such as CEA, NSE, CA19-9 and CA125 before and after treatment, as well as differences in levels of CD3+, CD4+, CD8+ and CD4+/CD8+ of T lymphocyte subsets between the two groups before and after treatment were compared and analyzed.

**Results:**

The total efficacy of the experimental group was 80%, which was significantly better than 57.5% of the control group (P=0.03). The incidence of adverse drug reactions in the experimental group was 17.5%, while that in the control group was 42.5%, showing a statistically significant difference (P=0.02), and the WBC count decreased more significantly in the control group (P=0.04). CEA, NSE, CA19-9 and CA125 decreased significantly in the experimental group after treatment compared with the control group, with a statistically significant difference (P=0.00). Moreover, the levels of CD3+, CD4+, CD4+/CD8+ in the experimental group after treatment were significantly higher than those in the control group, with statistically significant differences (CD3+, P=0.03; CD4+, P=0.00; CD4+/CD8+, P=0.00), while CD8+ did not change significantly (P=0.95).

**Conclusions:**

Highly agglutinative staphylococcin combined with chemotherapy is a safe and effective treatment regimen with definite curative effect for patients with intermediate or advanced oral cancer. With such a regimen, tumor markers are remarkably reduced, immune function can be significantly improved, and adverse reactions will be evidently reduced.

## INTRODUCTION

Oral cancer is a common disease in the department of stomatology, including lip cancer, tongue cancer, buccal cancer, gingival cancer, oral floor cancer, hard palate cancer, etc.^1^ Oral cancer is dominated by squamous cell carcinoma, which, as one of the most common and invasive cancers that invade local tissues, may cause metastasis and have a high mortality.^2^ Currently, surgery and chemoradiotherapy are the preferred treatment methods for oral squamous cell carcinoma in clinical practice. However, patients with intermediate or advanced oral cancer usually have a tumor larger than 4cm^3^ and undergo a large range of surgical resection, resulting in severe impact on corresponding functions due to the destruction of the original anatomical structure.

Neoadjuvant chemotherapy, also known as preoperative induction chemotherapy, is an important treatment for locally advanced oral squamous cell carcinoma. It was shown in a study by Zhong et al.^4^ that neoadjuvant chemotherapy boasts of reducing the preoperative stage of patients, improving the long-term survival rate, and even achieving complete remission. However, treatment-related adverse reactions, such as nausea, vomiting, constipation, diarrhea, and oral mucositis, can be caused by chemotherapy during the treatment process. In severe cases, patients’ compliance with chemotherapy may be declined, and their body immunity may be reduced, seriously affecting the efficacy of chemotherapy.^5^

Highly agglutinative staphylococcin (HAS), a super antigen derived from the metabolites of Staphylococcus aureus, has been proven to inhibit and kill tumors, repair tissues and cells, increase white blood cell count, and improve immune function.^6^ In this study, HAS combined with neoadjuvant chemotherapy was used to treat patients with intermediate or advanced oral cancer, and remarkable clinical effects were achieved.

## METHODS

This retrospective analysis was performed on 80 patients with intermediate or advanced oral cancer admitted to Affiliated People’s Hospital of Fujian University of Traditional Chinese Medicine from January 2020 to January 2022. They were divided into two groups according to the treatment they were treated with: the control group and the experimental group, with 40 cases in each group. Among them, there were 27 males and 13 females in the experimental group, aged 55-72 years with an average of 62.70±5.49 years, and 26 males and 14 females in the control group, aged 57-74 years with an average of 63.12±5.37 years. No significant difference can be seen in the comparison of general data between the two groups, which was comparable between the two groups (Table-I).

**Table-I:** Comparative analysis of general data between the experimental group and the control group (*X̅*±*S*) n=40.

Indicators	Experimental group	Control group	t/χ^2^	P
Age (years old)	62.70±5.49	63.12±5.37	0.35	0.73
Male (%)	27 (67.5%)	26 (65%)	0.06	0.81
TNM staging				
III	23 (57.5%)	21 (52.5%)	0.20	0.65
IV	17 (42.5%)	19 (47.5%)	0.23	0.65
Tumor location				
Lip	17 (42.5%)	18 (45%)	0.05	0.82
Tongue	11 (27.5%)	10 (25%)	0.06	0.80
Gum	9 (22.5%)	7 (17.5%)	0.31	0.58
Other	3 (7.5%)	5 (12.5%)	0.56	0.46
Tumor diameter (cm)	4.67±0.33	4.79±0.62	1.08	0.28

P>0.05.

### Ethical Approval

The study was approved by the Institutional Ethics Committee of Affiliated People’s Hospital of Fujian University of Traditional Chinese Medicine on January 20, 2020 (No.[2020]11), and written informed consent was obtained from all participants.

### Inclusion criteria:- -

- Patients who met the diagnostic criteria of oral cancer and were found to have accurately measurable masses by imaging;^7^
- Patients with typical clinical symptoms such as dysphagia, bleeding, masses, etc.;
- Patients with intermediate or advanced clinical stages (UICC, Stage III-IV);^8^
- Patients with KPS score ≥60 points;
- Patients in good general condition and able to tolerate chemotherapy
- Patients with an expected survival of ≥6 months;
- Patients without distant metastasis of tumor;
- Patients who have good compliance with their own treatment and signed an informed consent; Patients younger than 75 years old who had not received initial treatment such as radiotherapy or chemotherapy.

### Exclusion Criteria:

- Patients with tumors at other sites;
- Patients with severe organic diseases of heart, liver and kidney or congenital diseases;
- Patients who have received previous radiotherapy or chemotherapy;
- Patients with abnormal mental or cognitive function and unable to cooperate with the implementation of this study;
- Patients with co-existing diseases that affected the results of the study, such as autoimmune diseases and inflammatory diseases
- Patients who have recently taken relevant drugs that affect the study, such as immunosuppressants and hormones;
- Patients with poor physical fitness, cachexia, and unstable vital signs;
- Patients allergic to the drugs involved in the study.

Patients in the two groups were subjected to blood cell analysis, liver function, and renal function tests, and the abnormal indicators were corrected accordingly. During the treatment, nutritional assessment was performed, nutritional support treatment was given to patients with malnutrition, while basic treatments such as antiemetic correction of electrolyte disorder were given to those with corresponding symptoms.

Patients in the control group were given paclitaxel combined with cisplatin chemotherapy regimen: paclitaxel 150 mg/m2 and cisplatin 100 mg/m2 were hydrated on the first day of chemotherapy, with 28 days as one chemotherapy cycle for a total of 3 cycles of treatment. Patients in the experimental group received intramuscular injection of 500U highly agglutinative staphylococcin once a day for two weeks on the basis of chemotherapy, and continued the next course of treatment after one week of withdrawal, with a total of two courses.

### Observation Indicators:

Efficacy evaluation: All patients were evaluated according to solid tumor efficacy evaluation criteria 1.0 (RECIST1.0) after treatment:^9^ Complete response (CR): complete disappearance of lesions; Partial response (PR): a reduction of more than 30% in the sum of measured diameters of the target lesion relative to baseline; Stable disease (SD): a 20%-30% reduction in the maximum diameter of the lesion; Progression disease (PD): an increase of at least 20% in the sum of the long diameters of all target lesions, with an absolute increase of more than 5mm; Or the appearance of new lesions. Total response rate = (CR+PR) number of cases/total number of cases×100%;

Adverse drug reaction evaluation: Adverse drug reactions occurred in the two groups within one month after medication were recorded, including rash, gastrointestinal reactions, oral mucositis, leucopenia, neuritis, liver function damage and so on;

Comparative analysis of tumor markers: Fasting blood was taken in the morning before and after treatment, respectively, to detect CEA, NSE, CA19-9, CA125 and other tumor markers, and the differences between the two groups were compared and analyzed;

Analysis of immune status: Fasting blood was taken in the morning before and after treatment, respectively, to detect CD3+, CD4+, CD8+, CD4+/CD8+ levels of T lymphocyte subsets, and the differences between the two groups before and after treatment were compared and analyzed.

### Statistical Analysis

All the data were statistically analyzed by SPSS 20.0 software, and the measurement data were expressed as (*X̅*±*s*). Two independent sample t-test was used for inter-group data analysis, paired t test was used for intra-group data analysis, and χ^2^ was adopted for rate comparison. P<0.05 indicates a statistically significant difference.

## RESULTS

The comparative analysis of the treatment effect of the two groups is shown in Table-II, indicating that the total response rate of the two groups after treatment was 80% in the experimental group, which was significantly better than that of the control group (57.5%), showing a statistically significant difference (P=0.03).

**Table-II:** Comparative analysis of treatment effect between the two groups (*X̅*±*S*) n=40.

Group	CR	PR	SD	PD	Total response rate
Experimental group	8	24	5	3	32 (80%)
Control group	6	17	12	5	23 (57.5%)
χ^2^					4.71
P					0.03

P<0.05.

The comparative analysis of the incidence of adverse drug reactions between the two groups of patients after treatment is shown in Table-III, indicating that the incidence of adverse drug reactions in the experimental group was 17.5%, which was significantly lower than the 42.5% in the control group, showing a statistically significant difference (P=0.02), and the WBC count decreased more significantly in the control group (P=0.04) (Table-III).

**Table-III:** Comparative analysis of adverse drug reactions between the two groups after treatment (*X̅*±*S*) n=40.

Group	Skin rash	Gastrointestinal reaction	Stomatitis	WBC reduction	Neuritis	Liver damage	Incidence
Experimental group	1	1	2	0	2	1	7 (17.5%)
Control group	2	3	3	4	3	2	17 (42.5%)
χ^2^							5.95
P							0.02

P<0.05.

No significant difference was observed in the levels of CEA, NSE, CA19-9, and CA125 before treatment between the two groups (P>0.05). After treatment, all the above indicators were lower than before treatment. CEA, NSE, CA19-9 and CA125 in the experimental group were significantly lower than those in the control group, with statistically significant differences (P=0.00) (Table-IV).

**Table-IV:** Comparative analysis of tumor marker levels between the two groups before and after treatment (*X̅*±*S*) n=40.

Indicators	Observation points	Experimental group	Control group	t	p
CEA ( ng/ml)	Before treatment	3.08±0.73	2.97±0.39	0.84	0.40
	After treatment*	1.76±0.15	2.28±0.60	5.32	0.00
NSE ( ng/ml)	Before treatment	2.78±0.32	2.86±0.30	1.01	0.32
	After treatment*	1.27±0.24	1.76±0.85	3.51	0.00
CA19-9 ( kU/L)	Before treatment	23.74±7.73	23.39±6.97	0.21	0.83
	After treatment*	14.32±4.31	18.30±5.54	3.60	0.00
CA125 ( U/ml)	Before treatment	43.46±6.82	42.71±6.59	0.50	0.62
	After treatment*	23.35±5.41	28.42±5.26	4.25	0.00

* p<0.05

No significant difference was observed in the levels of CD3+, CD4+, CD8+, CD4+/CD8+ between the two groups before treatment (P>0.05). The levels of CD3+, CD4+, CD4+/CD8+ in the experimental group after treatment were significantly higher than those in the control group, with statistically significant differences (CD3+, P=0.03; CD4+, P=0.00; CD4+/CD8+, P=0.00), while CD8+ did not change significantly (P=0.95) (Table-V).

**Table-V:** Comparison of T lymphocyte subsets between the two groups before treatment (*X̅*±*S*) n=30.

Indicators	Observation points	Experimental group	Control group	t	p
CD3+ (%)	Before treatment	41.77±6.25	41.83±6.47	0.04	0.96
	After treatment*	47.32±6.80	43.98±6.42	2.26	0.03
CD4+ (%)	Before treatment	26.53±4.07	26.36±4.71	0.17	0.86
	After treatment*	35.66±5.42	30.32±5.70	4.29	0.00
CD8+ (%)	Before treatment	23.86±4.29	24.04±3.59	0.21	0.83
	After treatment*	23.75±4.83	23.73±4.51	0.02	0.95
CD4+/CD8+	Before treatment	1.39±0.25	1.41±0.23	0.37	0.71
	After treatment*	1.79±0.26	1.58±0.40	2.78	0.00

* p<0.05.

## DISCUSSION

Oral cancer is commonly seen in middle-aged and elderly people over 40 years old, with the characteristics of high malignancy, rapid progress, as well as easy invasion of adjacent tissues and lymph nodes. Most patients with oral cancer are in intermediate or advanced stage as soon as they are diagnosed, with extremely poor prognosis.^10^ Oral squamous cell carcinoma (OSCC) has been considered to be the most common malignancy of the head and neck region of patients worldwide.^11^ Oral cancer is currently unclear in its pathogenesis, which is generally considered to be related to various factors such as smoking, drinking, regular consumption of betel nut and other bad habits, genetic factors, nutritional status and so on. Repeated stimulation of gingival and oral mucosa by long-term toxicity and irritant substances produced by smoking and drinking makes oral cavity in a stress state, which leads to chronic inflammation of oral mucosa and activation of potential canceration.^12^ Most patients with oral cancer in developing countries are in advanced stage due to limitations of medical conditions.^13^ Despite its superficial location, oral cancer is easily detected and diagnosed clinically, and ironically, most patients with oral cancer are locally advanced, including all those with stage III/IV tumors without distant metastasi.^14^

Combination treatment options such as surgery combined with radiotherapy and chemotherapy are the preferred treatment options for clinical treatment of intermediate or advanced oral cancer.^15^ Among them, chemotherapy has always been an important means of treatment, which can significantly reduce the tumor burden in a short time, thereby effectively alleviating the clinical symptoms of patients and controlling the progress of the disease. It was considered by Alzahrani et al.^16^ that chemotherapy could be used to replace surgery as curative treatment or neoadjuvant therapy to promote surgical effect in the case that many advanced patients could not achieve complete surgical resection. Kim et al.^17^ believed that some patients in the intermediate or advanced stage have a low possibility of being cured and surgical removal of the tumor. However, chemotherapy allows the tumor to alter its aggressiveness and potentially preserve organ function while increasing tumor respectability. The median OS of patients who underwent resection was significantly superior to that of patients without preoperative chemotherapy.^18^ A 20-year follow-up study indicated that chemotherapy is a feasible strategy for local organ preservation in patients with locally advanced OC-SCC (Oral Squamous Cell Carcinoma).^19^ Paclitaxel can interfere with the polymerization of microtubules and inhibit the mitosis of tumor cells, with high anti-cancer activity. It is often used clinically in the treatment of solid tumors such as ovarian cancer and cervical cancer.^20^ Cisplatin may exert a broad-spectrum anti-cancer effect by inhibiting DNA replication and synthesis.^21^

However, chemotherapeutic drugs can not only kill tumor cells, but also cause damage to normal tissue cells, leading to a series of chemotherapy-related side effects. In such a case, patients suffer from loss of appetite, decreased immunity, and reduced compliance, resulting in unsatisfactory treatment effects.^22^ In particular, elderly patients with locally advanced oral cancer have similar remission rates and survival rates compared with young patients during chemotherapy, but may suffer from higher treatment-related toxicity and immunosuppression, resulting in chemotherapy intolerance or non-cooperation.^23^ Highly agglutinative staphylococcin (HAS), a mixture of staphylococcus aureus culture filtrates, has been used clinically as an immunomodulator in the treatment of a variety of tumors. It was believed by Yu et al.^24^ that HAS combined with chemotherapy in the treatment of patients with advanced breast cancer is touted as significantly improving patients’ WBC count, increasing their immunity and appetite, which is extraordinarily beneficial to the recovery of patients. Gu et al.^25^ believed that HAS could improve the immune function of patients and inhibit tumor growth. It was also confirmed in an animal experiment by Mu et al.^26^ that HAS could significantly improve the immune status of rabbits after joint surgery and ameliorate the function of T lymphocytes.

It was confirmed in our study that the total response rate of patients with intermediate or advanced oral cancer treated with HAS combined with neoadjuvant chemotherapy was significantly higher than that treated with chemotherapy alone (80% VS 57.5%), with a statistically significant difference (P=0.03). The incidence of adverse drug reactions in the experimental group was 17.5%, while that in the control group was 42.5%, showing a statistically significant difference (P=0.02), and the WBC count decreased more significantly in the control group (P=0.04). CEA, NSE, CA19-9 and CA125 decreased significantly in the experimental group after treatment compared with the control group, with a statistically significant difference (P=0.00). Moreover, the levels of CD3+, CD4+, CD4+/CD8+ in the experimental group after treatment were significantly higher than those in the control group, with statistically significant differences (CD3+, P=0.03; CD4+, P=0.00; CD4+/CD8+, P=0.00).

### Limitations of this study

It includes a small number of samples were included, and follow-up and operation-related indicators were not included. With the increase of the sample size, positive measures will be taken to further improve the operation related indicators and increase follow-up content, so as to conduct a more objective evaluation of the effect of the treatment regimen on surgery and long-term effect and other observation indicators.

## CONCLUSION

Highly agglutinative staphylococcin (HAS) combined with chemotherapy is a safe and effective treatment regimen with definite curative effect for patients with intermediate or advanced oral cancer. With such a regimen, tumor markers are remarkably reduced, immune function can be significantly improved, and adverse reactions will be evidently reduced.

### Authors’ Contributions:

**NG:** Designed this study, prepared this manuscript, are responsible and accountable for the accuracy and integrity of the work.

**XS:** Collected and analyzed clinical data. **GL:** Data analysis, significantly revised this manuscript.
